# Association between the occurrence of adenomyosis and the clinical outcomes of vaginal repair of cesarean section scar defects: an observational study

**DOI:** 10.1186/s12884-022-04529-x

**Published:** 2022-03-08

**Authors:** Huihui Chen, Wenjing Wang, Husheng Wang, Xipeng Wang

**Affiliations:** 1 Department of Obstetrics and Gynecology, Xin Hua Hospital affiliated to Shanghai Jiao Tong University School of Medicine, 1665 Kong Jiang Road, Yangpu District, Shanghai, 200092 China; 2 Department of Radiology, Shanghai First Maternity and Infant Hospital, Tongji University School of Medicine, Shanghai, 201204 China

**Keywords:** Adenomyosis, Cesarean section, Menstrual disorders, Surgery

## Abstract

**Background:**

To examine the correlation between the occurrence of adenomyosis and the outcome of vaginal repair of cesarean section scar defects (CSDs).

**Methods:**

A total of 278 women with CSD were enrolled in this retrospective observational cohort study at the Shanghai First Maternity & Infant Hospital between January 2013 and August 2017. Patients were divided into two groups according to preoperative magnetic resonance imaging (MRI) findings: the adenomyosis group and the non-adenomyosis group. They all underwent vaginal excision and suturing of CSDs and were required to undergo examinations 3 and 6 months after surgery. Preoperative and postoperative clinical information was collected. Optimal healing was defined as a duration of menstruation of no more than 7 days and a thickness of the residual myometrium (TRM) of no less than 5.8 mm after vaginal repair.

**Results:**

Before vaginal repair, for patients in the adenomyosis group, the mean duration of menstruation was longer and TRM was significantly thinner than those in patients in the non-adenomyosis group (*p* < 0.05). The TRM and duration of menstruation 3 and 6 months after surgery were significantly improved in both groups (*p* < 0.05). There were more patients with optimal healing in the non-adenomyosis group than in the adenomyosis group (44.7% vs. 30.0%; *p* < 0.05). Furthermore, 59.3% (32/54) of the women tried to conceive after vaginal repair. The pregnancy rates of women with and without adenomyosis were 66.7% (8/12) and 61.9% (26/42), respectively. The duration of menstruation decreased significantly from 13.4 ± 3.3 days before vaginal repair to 7.6 ± 2.3 days after vaginal repair in 25 patients (*p* < 0.001). The TRM increased significantly from 2.3 ± 0.8 mm before vaginal repair to 7.6 ± 2.9 mm after vaginal repair (*p* < 0.001).

**Conclusions:**

Vaginal repair reduced postmenstrual spotting and may have improved fertility in patients with CSDs. Patients with adenomyosis are more likely to have suboptimal menstruation and suboptimal healing of CSDs. Adenomyosis might be an adverse factor in the repair of uterine incisions.

## Introduction

The World Health Organization (WHO) suggested that the rate of cesarean sections be maintained at 15% [1]. However, in China, the rate of cesarean section increased from 28.8% in 2008 to 34.9% in 2014, and in 2018 reached 36.7% [2]. With the increase in the number of cesarean sections, cesarean section scar defects (CSDs), as a new type of iatrogenic disease, have gained enormous research momentum. CSDs were first described by Morris in 1995 as a pouch-like defect in the anterior uterine wall at the site of a previous cesarean section [3]. Many patients with CSD are asymptomatic; however, many have reported intermenstrual spotting, dysmenorrhea, dyspareunia, and chronic pelvic pain. Other studies have reported that CSD is an adverse factor for uterine rupture and infertility [4–7].

Magnetic resonance imaging (MRI) and transvaginal sonography (TVS) are useful in the diagnosis of CSD, and both methods can determine the length, width, and depth of the defect and the thickness of the residual myometrium (TRM). In addition, MRI is useful in diagnosing other gynecological diseases such as fibroids, adenomyosis, ovarian tumors, and pelvic diseases.

Adenomyosis, as one of the manifestations of endometriosis that affects women of child-bearing age, is categorized by the presence of hypertrophic smooth muscle derived from ectopic endometrial glands and stroma within the myometrium [8, 9].

Vaginal repair due to CSDs is a minimally invasive and effective method that maintains fertility [10–12]. Patients suffering from intermittent postmenstrual bleeding who underwent vaginal repair still had CSDs, although the size of the defect and the clinical symptoms were improved significantly. In another study, adenomyosis was reported to involve repeated autotraumatization and self-healing of the endometrial-myometrial junctional zone, thereby affecting myometrium healing [13]. This prompted us to examine the factors involved in the less-than-optimal outcome of vaginal repair.

Here, we hypothesized that adenomyosis might be an adverse factor for uterine repair. We retrospectively reviewed the MRI findings of patients with CSDs to determine whether there is a correlation between the occurrence of adenomyosis and the outcomes of vaginal repair. We also provide clinical recommendations for the treatment of CSDs.

## Patients and methods

This retrospective study was approved by the Ethics Committee of the Shanghai First Maternity & Infant Hospital (KS1512). We retrieved data by diagnostic codes from outpatients with CSDs who underwent MRI to determine the length, width, and depth of the defect and subsequent vaginal surgery at the Tongji University-affiliated Shanghai First Maternity & Infant Hospital from January 2013 to August 2017. All MRI scans were re-evaluated by an experienced radiologist. After educating the patients on the advantages and disadvantages of vaginal surgery, they provided written informed consent. According to the findings of preoperative MRI scans, the patients were divided into two groups: the adenomyosis group and the non-adenomyosis group.

**Fig. 1:**
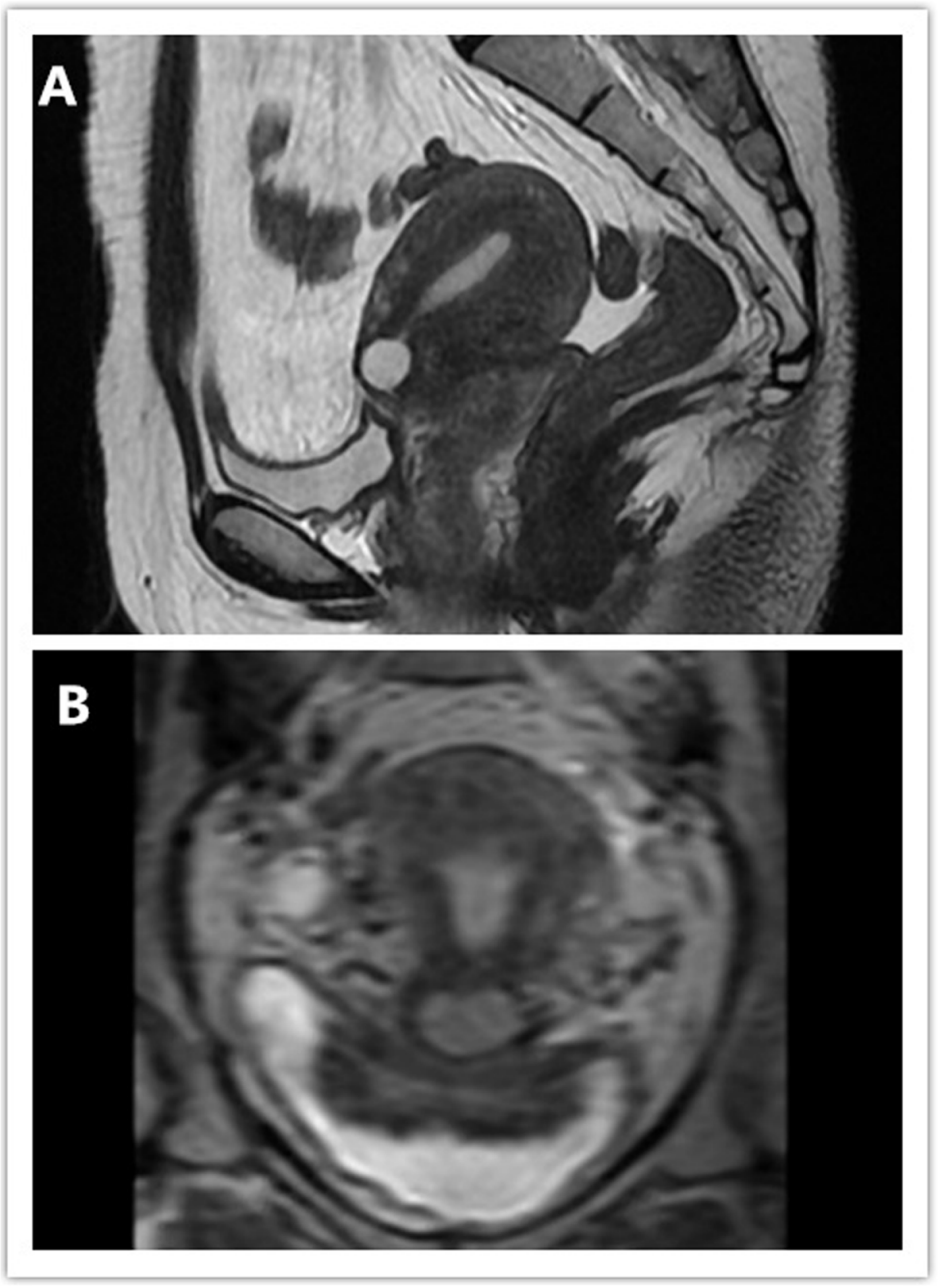
MRI images of cesarean scar defects. **A.** Sagittal view on T2 images. **B.** Coronal view on T2 images

**Fig. 2:**
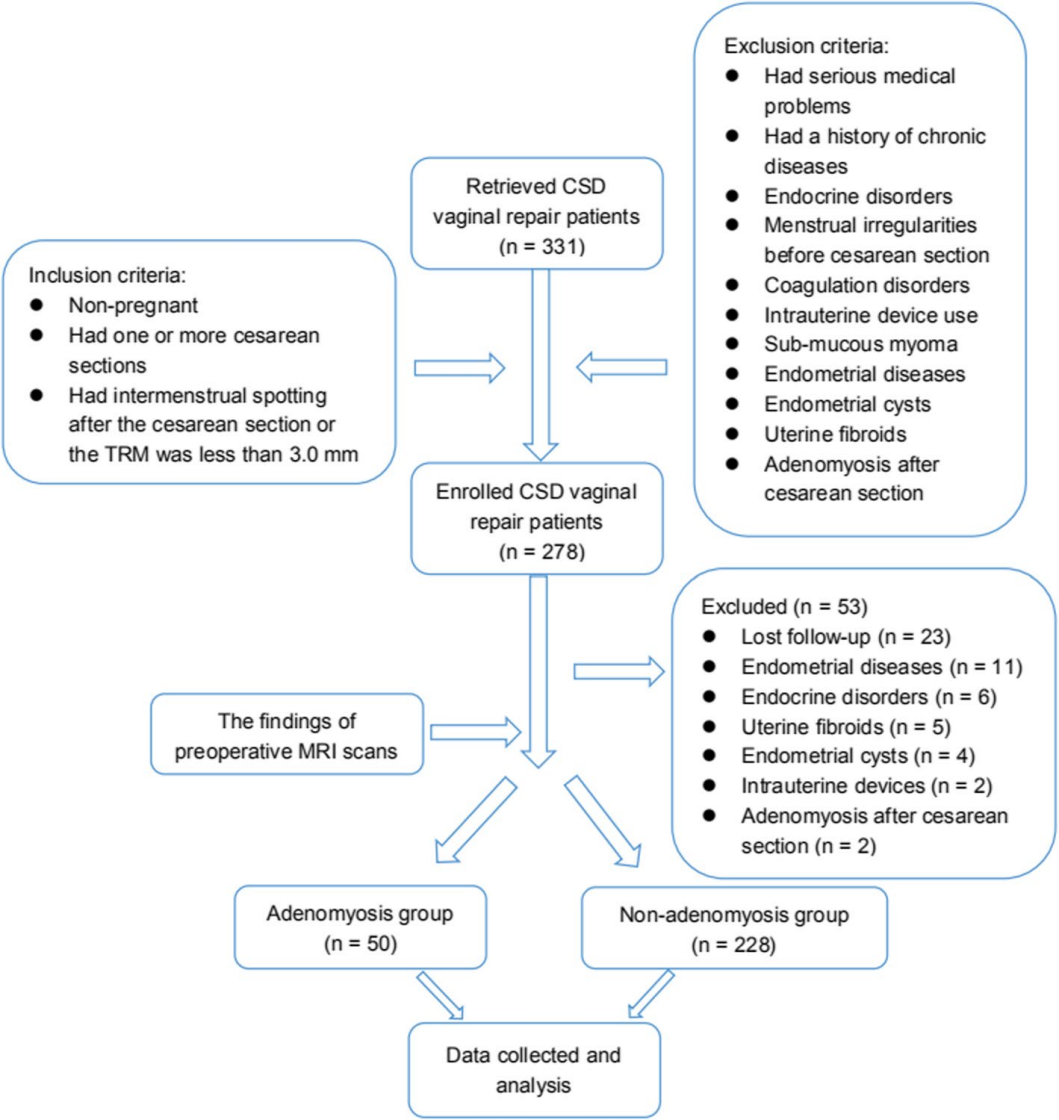
Flow chart of study

The inclusion criteria were nonpregnant patients who had one or more cesarean sections, patients who had intermenstrual spotting after cesarean section, patients in which the TRM was less than 3.0 mm at the preoperative stage, and patients who underwent MRI and TVS to evaluate the size of the defect and TRM before surgery [14] (Fig. 1). All patients had no serious medical problems (important visceral function in the normal range). Patients who had a history of chronic diseases (such as cerebrocardiovascular diseases, malignancies and diabetes mellitus), endocrine disorders, menstrual irregularities before cesarean section, coagulation disorders, intrauterine device use, submucous myoma, endometrial diseases, endometrial cysts, uterine fibroids, or adenomyosis after cesarean section were excluded from this study (Fig. 2).

### Surgical procedures

**Fig. 3:**
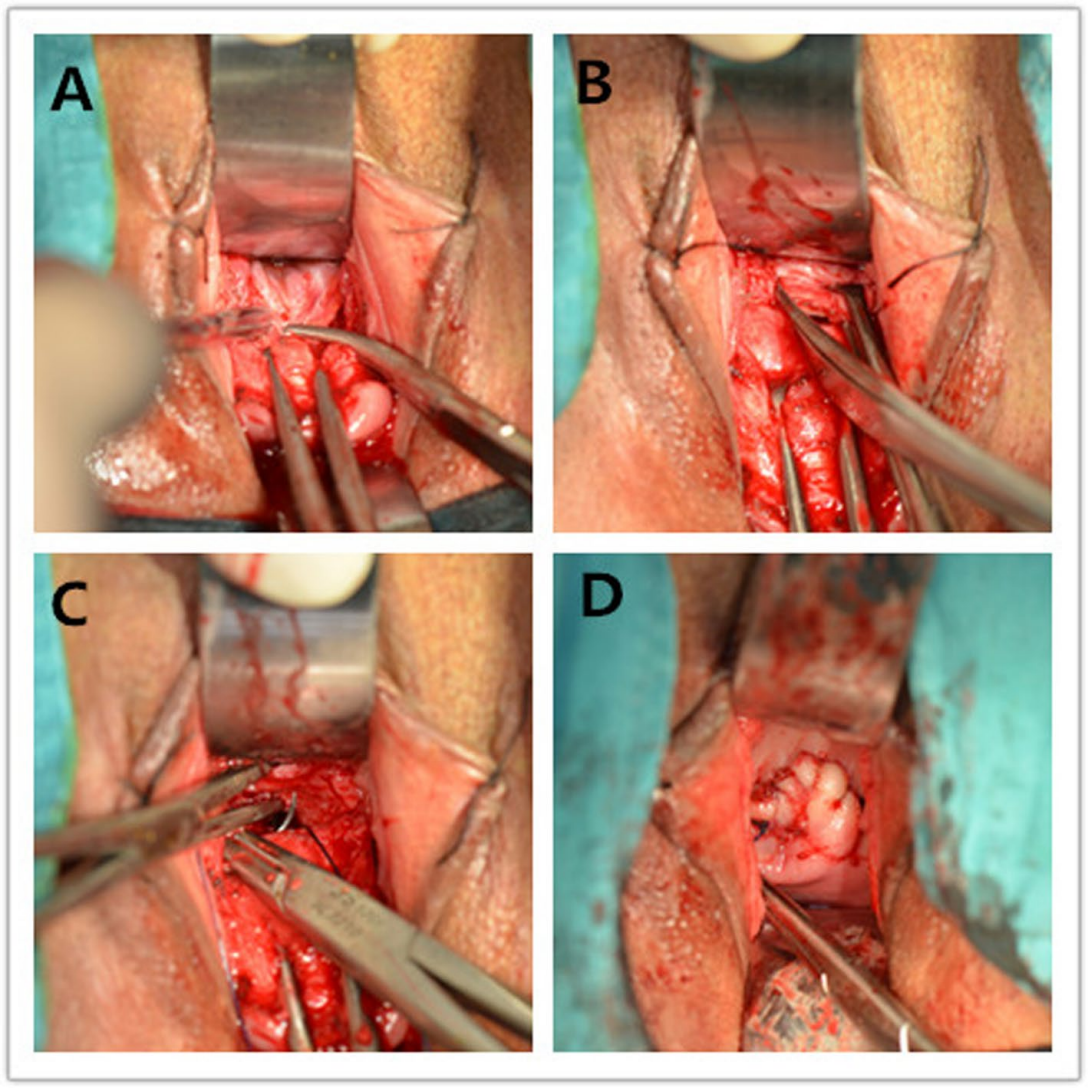
Transvaginal surgery procedure. **A.** The opening of the anterior peritoneal reflection; **B,** the trimming of the CSD edge; **C,** the closing of the myometrium; and **D,** the end of the procedure

All surgical procedures were performed by an experienced surgeon as previously described [10, 11, 15]. After administering continuous epidural anaesthesia, the patients were placed in the bladder lithotomy position. All patients had empty bladders. The anterior peritoneal reflection was opened, and the abdominal cavity was entered. After exposing the lower uterine segment, a probe was used to identify the CSD area. The tissue was trimmed with scissors to reveal the healthy myometrium, and the CSD tissue was completely removed. The myometrium was closed using a double-layer closure of 1–0 absorbable sutures with an interrupted suture (Fig. 3).

### Magnetic resonance imaging

MRI scans were conducted with a 1.5 T MR scanner (Optima MR360; General Electric Company, USA). The patients underwent routine screening of the pelvic sagittal and coronal planes and the fat-suppressed sagittal and coronal planes. All images were evaluated by an experienced radiologist. Several baseline characteristics were assessed on T2-weighted images, including the position of the uterus (anteverted or retroverted), the diameter of the CSD (the length, width, and depth), the TRM, and the presence of adenomyosis, endometriosis, or uterine fibroids.

**Fig. 4:**
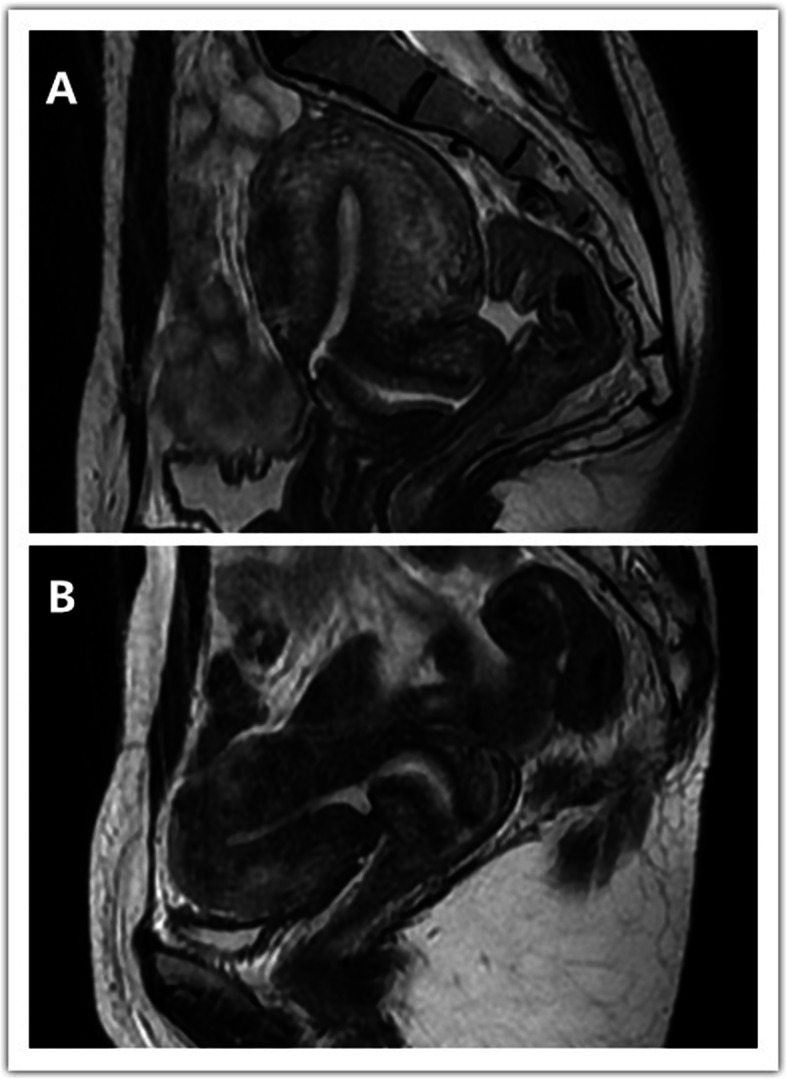
MRI scans of cesarean scar defects with adenomyosis. **A.** Sagittal view on T2 images (retroflexed uterus). **B** Sagittal view on T2 images (anteflexed uterus)

The main features of adenomyosis were an increased thickness of the junctional zone of the uterus (exceeding 12 mm) and the presence of intramyometrial cyst(s) or a heterogeneous myometrium, which were associated with heterogeneously hyperintense regions on T2-weighted and sometimes T1-weighted images (Fig. 4).

### Data collection and follow-up

Data were identified using the diagnostic codes (N85.814) in billing records. Preoperative and postoperative clinical information was collected from medical files, including the following: age; other general patient details; number of cesarean sections; history of menstrual conditions; position of the uterus; hemoglobin level on the first postoperative day; length of hospitalization; hospitalization cost; CSD length, width, and depth; and the TRM. All patients were required to undergo examinations 3 and 6 months after surgery to obtain information on menstruation and to measure anatomical data after surgery (the TRM) based on MRI or TVS. Patients who failed to return in a timely manner were followed-up by telephone and reminded to complete the measure as soon as possible. Long-term follow-up was conducted in patients with subsequent pregnancy attempts. Data on gestational age, neonatal birth weight, infant Apgar score and pregnancy complications were collected from the patients by telephone and medical records. Optimal healing was defined as a duration of menstruation of no more than 7 days and a TRM of no less than 5.8 mm after vaginal repair [16].

### Statistical analysis

SPSS 22.0 software (SPSS Inc., Chicago, IL, USA) was used for all statistical analyses. Data are presented as the means ± SD or percentages as appropriate. A paired *t-test* was used to analyze the preoperative and postoperative data. Continuous data are presented as medians and ranges, and categorical data are presented as frequencies and percentages. The hospitalization length and cost were analyzed using the Mann-Whitney U test. Categorical variables were analyzed using the chi-squared test or Fisher’s exact test when the number of variables was less than five. *P-values* < 0.05 were considered to be statistically significant.

## Results

### Patient characteristics

The pre-treatment demographic data are summarized in Table 1. Data from a total of 331 patients were retrieved in this study. Fifty-three patients were excluded. Twenty-three patients were lost to follow-up, eleven patients had endometrial diseases, six patients had endocrine disorders, five patients had uterine fibroids, four patients had endometrial cysts, two patients had intrauterine devices and two patients had adenomyosis after cesarean section. In total, 278 patients were enrolled and divided into the adenomyosis group (*n* = 50), in which the mean patient age was 32.6 ± 3.8 years, and the non-adenomyosis group (*n* = 228), in which the mean patient age was 32.8 ± 3.6 years (Fig. 2). No significant differences were observed between the groups in the number of cesarean sections, the duration of postmenstrual spotting before cesarean section, the mean preoperative CSD length, width, and depth, or the TRM measured by TVS (*p* > 0.05). However, the duration of postmenstrual spotting after cesarean section in the adenomyosis group was significantly longer than that in the non-adenomyosis group (15.3 ± 4.1 days versus 14.0 ± 3.2 days, *p* < 0.05). In addition, the mean preoperative width was significantly longer and the TRM was thicker in the adenomyosis group than that in the non-adenomyosis group (15.0 ± 3.7 mm versus16.6 ± 4.4 mm; 2.9 ± 1.1 mm versus 2.5 ± 1.2 mm, *p* < 0.05).

**Table 1:** Patient baseline characteristics prior to treatment

Demographic	Adenomyosis group(*n* = 50)	Non-adenomyosis group (*n* = 228)	*P* value
Age (y)	32.6 ± 3.8 (23–41)	32.8 ± 3.6 (23–42)	0.735
Gravidity (n)	2.2 ± 1.1 (1–5)	2.0 ± 1.1 (1–6)	0.175
Number of cesarean deliveries (n)	1.4 ± 0.5 (1–3)	1.3 ± 0.5 (1–3)	0.089
Duration of menstruation before cesarean delivery (d)	6.3 ± 1.3 (3–10)	6.2 ± 1.1 (3–9)	0.793
Duration of postmenstrual spotting after cesarean delivery (prior to surgical repair of CSD) (d)	15.3 ± 4.1 (5–25)	14.0 ± 3.2 (5–30)	0.013
Uterus position			
anteflexion	25 (50.0%)	99 (43.4%)	0.244
retroflexion	25 (50.0%)	129 (56.6%)	
TVS findings (mm)			
CSD length	7.7 ± 3.3 (2.0–17.0)	8.0 ± 3.5 (2.0–18.0)	0.640
CSD width	12.2 ± 4.4 (3.0–23.0)	12.3 ± 5.7 (3.0–30.0)	0.911
CSD depth	7.1 ± 3.4 (2.0–19.0)	6.4 ± 2.8 (2.0–18.0)	0.177
TRM	2.9 ± 1.4 (1.0–9.0)	2.7 ± 1.1 (0.7–7.0)	0.253
MRI findings (mm)			
CSD length	9.3 ± 3.8 (1.0–18.5)	9.1 ± 3.2 (1.0–20.0)	0.653
CSD width	15.0 ± 3.7 (5.0–22.4)	16.6 ± 4.4 (5.0–28.4)	0.018
CSD depth	6.0 ± 2.0 (2.5–11.3)	6.2 ± 2.6 (1.6–21.0)	0.619
TRM	2.9 ± 1.1 (1.0–6.0)	2.5 ± 1.2 (0.5–10.1)	0.033

Data presented as mean ± SD (range) except for uterus position. Data presented as numbers (percentage) for uterus position

*CS* caesarean section, *CSD* cesarean scar defect, *TRM* thickness of the residual myometrium, *TVS* transvaginal sonography

### Clinical outcomes after surgery

All patients underwent vaginal repair. The clinical data are summarized in Table 2. No significant differences in the duration of the surgical procedure, hospitalization stay, or hospitalization cost were observed between the groups (*p* > 0.05). In addition, four out of the 228 patients in the non-adenomyosis group had complications (two cases of bladder injury and two cases of hematoma), whereas one out of the 50 patients in the adenomyosis group had a complication (hematoma). Thus, the incidence of perioperative complications was 1.8 and 2.0% in the two groups, respectively.

**Table 2:** Clinical outcomes after treatment for cesarean scar defect

Variable	Adenomyosis group (*n* = 50)	Non-adenomyosis group (*n* = 228)	*P* value
Hemoglobin on the first postoperative day (g/L)	99.5 ± 13.4 (74.2–125.0)	106.0 ± 11.2 (77.2–134.0)	0.012
Blood loss during operation (ml)	31.8 ± 20.0 (10–100)	30.8 ± 23.6 (10–200)	0.745
Duration of surgical procedure (min)	57.0 ± 11.8 (30–90)	55.9 ± 9.4 (25–99)	0.497
Length of hospital stay (d)	7.0 (1.0)	6.5 (1.0)	0.296^**^
Hospitalization cost (CNY)	10,870 (2175.3)	11,085.0(1997.3)	0.528^**^
Complications (n)			
Bladder injury	0 (0.0%)	2 (.9%)	0.672*
Hematoma	1 (2.0%)	2 (.9%)	0.450*

Data presented as mean ± SD (range) except for complications, length of hospital stay and hospitalization cost, where complications presented as numbers (percentage) and length of hospital stay and hospitalization cost presented as median (interquartile range)

* Fisher’s Exact Test was used. ^**^ Mann-Whitney U Test was used

### Gynecological follow-up

Data on the duration of menstruation and the TRM before and after surgery are summarized in Table 3. The mean durations of menstruation and TRM of all the patients were significantly improved than those before surgery (*p* < 0.05). Similarly, for the non-adenomyosis group, the mean durations of menstruation at the 3- and 6-month follow-ups were significantly shorter than those before surgery (8.1 ± 2.5 days and 8.3 ± 2.4 days, respectively, *p* < 0.05). For the adenomyosis group, the mean durations of menstruation at the 3- and 6-month follow-ups were significantly shorter than those before surgery (*p* < 0.05). The TRM at the median-month follow-up was significantly strengthened in both groups (*p* < 0.05).

**Table 3:** Duration of menstruation and TRM before surgery and at 3, 6 and median months after surgery

		Number of patients	All patients	*P* value^*^	Adenomyosis group	*P* value^*^	Non-adenomyosis group	*P* value^*^
Duration of menstruation	Before surgery	231	14.3 ± 3.4 (5–30)	< 0.001	15.4 ± 4.1 (5–20)	< 0.001	14.1 ± 3.2 (5–30)	< 0.001
	At 3 months		8.1 ± 2.4 (3–18)		8.1 ± 1.7 (5–12)		8.1 ± 2.5 (3–18)	
	Before surgery	191	14.4 ± 3.4 (5–30)	< 0.001	15.2 ± 4.1 (5–20)	< 0.001	14.2 ± 3.2 (5–30)	< 0.001
	At 6 months		8.3 ± 2.3 (4–15)		8.3 ± 2.0 (5–15)		8.3 ± 2.4 (4–15)	
TRM	Before surgery		2.7 ± 1.2 (0.5–10.1)	< 0.001	2.8 ± 1.1 (1.0–6.0)	< 0.001	2.4 ± 1.0 (0.5–10.1)	< 0.001
	At median months		7.4 ± 6.7 (1.0–12.0)		7.6 ± 2.8 (2.0–12.0)		7.3 ± 2.5 (1.0–12.0)	

Data presented as mean ± SD (range) for duration of menstruation and TRM before surgery and at 3, 6 and median months after surgery

^*^ The *p*-value compared the two time points (before surgery vs at 3 months, before surgery vs at 6 months, before surgery vs at median months) in each group

Data on the durations of menstruation at the 3- and 6-month follow-ups are summarized in Table 4. At the 3- and 6-month follow-ups, the mean durations of menstruation were 8.1 ± 2.3 days and 8.1 ± 1.6 days, respectively, and no significant difference was observed between the two groups (*p* > 0.05). Subsequently, we considered 7 days as the mean duration of menstruation and divided the patients into two subgroups. We found that 55.3% (126/228) of the patients in the non-adenomyosis group had an optimal duration of menstruation (≤7 days) at the 3- and 6-month follow-ups compared to 38.0% (19/50) of the patients in the adenomyosis group (*p* < 0.05). Optimal healing was more prevalent in the non-adenomyosis group than in the adenomyosis group (44.7% vs. 30.0%; *p* < 0.05).

**Table 4:** Comparison of follow-up data between two groups after treatment

Variable	Adenomyosis group (*n* = 50)	Non-adenomyosis group (*n* = 228)	*P* value
Duration of menstruation at median months after surgery	8.1 ± 1.6 (5–12)	8.1 ± 2.3 (3–16.5)	0.883
Duration of menstruation at median months after surgery			
≤ 7 days	19(38.0%)	126(55.3%)	0.029
> 7 days	31(62.0%)	102(44.7%)	
TRM (mm) by TVS at 3 months after surgery	7.9 ± 2.9 (2.0–12.0)	7.5 ± 2.4 (1.9–12.0)	0.460
TRM (mm) by MRI at 6 months after surgery	5.7 ± 2.9 (3.2–9.6)	4.8 ± 2.3 (1.2–9.9)	0.505
TRM at median months after surgery by MRI Staging	7.6 ± 2.8 (2.0–12.0)	7.3 ± 2.5 (1.0–12.0)	0.529
Optimal healing	15 (30.0%)	102 (44.7%)	0.038
Suboptimal healing	35 (70.0%)	126 (55.3%)	

Data presented as mean ± SD (range) for duration of menstruation and TRM at median months after surgery and TRM at 3 or 6 months after surgery. Data presented as numbers (percentage) for duration of menstruation at median months after surgery, TVS or MRI findings at 3 or 6 months after surgery and Class-A healing

*CSD* cesarean scar defect, *TVS* transvaginal sonography, *TRM* thickness of the residual myometrium

### Pregnancy follow-up

**Fig. 5:**
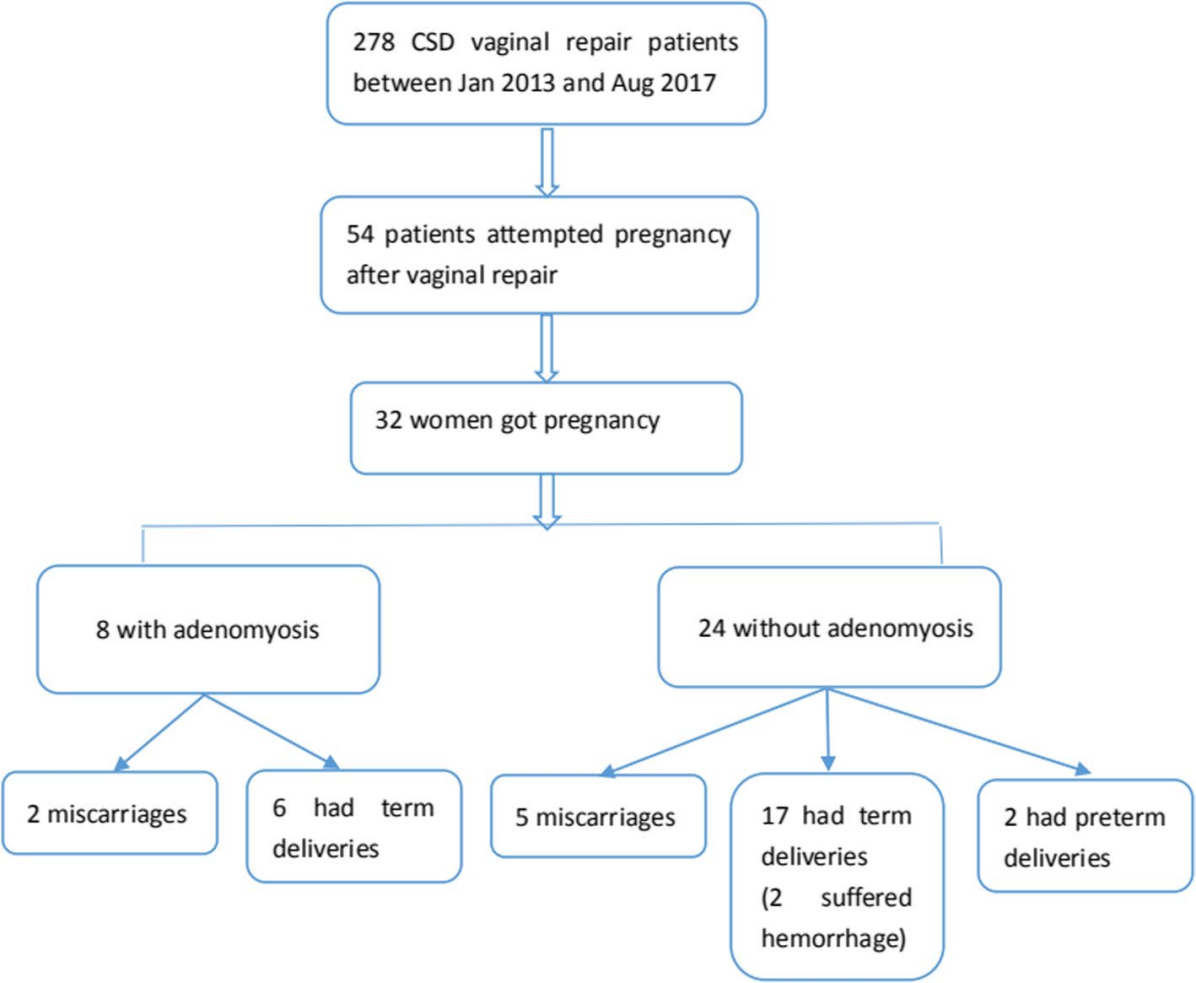
Obstetrical outcomes after vaginal repair of cesarean scar defects

The pregnancy outcome was assessed in 32 out of 54 women (59.3%) who attempted to conceive after vaginal repair (Fig. 5). Among these, there were 12 cases of adenomyosis and 42 cases of non-adenomyosis. For those who achieved pregnancy, the pregnancy rates of women with and without adenomyosis were 66.7% (8/12) and 57.1% (24/42), respectively. The data for 25 women (six with adenomyosis and 19 without adenomyosis) who achieved pregnancy and delivered infants are summarized in Table 5. By TVS, the TRM increased significantly from 2.3 ± 0.8 mm (range, 0.5–4.0 mm) to 7.6 ± 2.9 mm (range, 3.0–12.0 mm) at the 3-month follow-up after vaginal repair(*p* < 0.001). The duration of menstruation decreased significantly from 13.4 ± 3.3 days to 7.6 ± 2.3 days after vaginal repair (*p* < 0.001). All women selected cesarean section as the method of childbirth, and there were no cases of uterine rupture or dehiscence.

**Table 5:** Clinical characteristics of the women who achieved pregnancy without miscarriage

Demographic	Patients (*n* = 25)
Age (y)	31.0 ± 3.6 (27–38)
Number of cesarean deliveries (n)	1
Menstruation (d)	
Before VR	13.4 ± 3.3 (7–20)
After VR	7.6 ± 2.3 (4–14)
CSD size before VR (mm)	
CSD length	8.8 ± 3.0 (2.9–13.3)
CSD width	17.4 ± 5.0 (7.0–28.4)
CSD depth	6.3 ± 2.1 (2.7–10.2)
TRM	2.3 ± 0.8 (0.5–4.0)
Persistent CSD after VR, %	32.0 (8/25)
TRM after VR (mm)	7.6 ± 2.9 (3.0–12.0)
Preterm birth rate (%)	8.0 (2/25)
Neonatal birth weight (g)	3224.2 ± 401.0 (2400–4000)
Apgar score (5 min)	10
Postpartum hemorrhage rate (%)	8.0 (2/25)
adenomyosis rate (%)	24.0 (6/25)

Data were presented as the means ± SD or percentages

*CSD* cesarean scar defect, *VR* vaginal repair, *TRM* thickness of the residual myometrium

## Discussion

Results from our study showed that vaginal repair is a minimally invasive and effective method that maintains fertility in patients with CSD [10, 15, 17]. A total of 278 patients underwent pre- and postoperative MRI or TVS. We found that gynecological symptoms, such as postmenstrual spotting, and uterine morphology improved (Table 3). We also found that patients with adenomyosis were more likely to have suboptimal menstruation and suboptimal healing of CSDs. Adenomyosis might be an adverse factor in the repair of uterine incisions. To the best of our knowledge, the results regarding the association between the occurrence of adenomyosis and the clinical outcomes of vaginal repair of CSDs in nonpregnant women have not been published previously.

Adenomyosis is a common gynecological disease characterized by the infiltration of ectopic endometrial glands and/or stroma into the myometrium, thereby causing dysmenorrhea, pelvic pain, abnormal uterine bleeding, and infertility [8, 18, 19]. Fifty out of 278 patients (18.0%) had adenomyosis, which is consistent with previous studies reporting an incidence of 20% [20, 21]. The mean preoperative CSD width was smaller and the TRM was thicker in patients with adenomyosis than in patients without the disorder and this was due to the presence of hyperplastic and hypertrophic smooth muscle.

The duration of menstruation before cesarean section was longer in patients with adenomyosis than that in patients without the disorder; however, the results were not significantly different (*p* > 0.05). These patients suffered abnormal uterine bleeding after cesarean delivery. In addition, the duration of menstruation after cesarean section was significantly longer in patients with adenomyosis than in patients without the disorder (*p* < 0.05), suggesting that adenomyosis might disrupt the tissue repair process after cesarean section. In addition, after vaginal repair, CSD patients with adenomyosis had a more unfavorable prognosis. At follow-up, the duration of menstruation was optimal in patients with adenomyosis (*p* < 0.05). Furthermore, the optimal rate of optimal healing after vaginal repair was not achieved in patients with adenomyosis (Table 4), suggesting that adenomyosis was an adverse factor in the healing of uterine incisions.

Ectopic endometrial glands and the presence of stroma can cause repeated bleeding of the myometrium. Repeated tissue injury and repair caused by adenomyotic lesions increases the degree of fibrosis [13]. Ibrahim et al. reported the presence of myofibroblasts at the endometrial myometrial junctional zone in the uteri of patients with adenomyosis, suggesting that the tissue injury and repair mechanism was activated [22–24]. Repeated cycles of autotraumatization at the endometrial myometrial junctional zone can disrupt uterine muscular fibres, which eventually leads to endometrial basalis invagination and inhibits the healing process [13]. Therefore, damage to themyometrium in adenomyosis is not conducive to healing.

A total of 59.3% of the patients in our study achieved pregnancy after vaginal repair, with eight out of 12 women with adenomyosis achieving pregnancy, which was slightly higher than that in women without the disorder. Uterine rupture is a catastrophic complication during pregnancy and labor, especially for women with a history of cesarean section. The TRM is an indicator of uterine rupture or dehiscence, and although many risk factors can lead to these outcomes, there is an association between a thin TRM and uterine rupture or dehiscence [25]. However, the TRM cut-off remains controversial. It has been reported that the cut-off TRM value for the risk of uterine rupture should be set at 2.5–3.0 mm [4, 26, 27]. In this study, we found that the TRM of women who achieved pregnancy and delivered infants increased significantly from 2.3 ± 0.8 mm before surgery to 7.6 ± 2.9 mm after surgery, and the TRM was not less than 3 mm. Therefore, the pregnancy outcome was favorable, and there were no cases of uterine rupture or dehiscence. Furthermore, vaginal repair not only reduced menstrual spotting but also reconstructed the uterus to preserve fertility in patients with CSDs.

There were several limitations in this study. First, our study was a single- center retrospective study, although the sample size was fairly large. Second, information on the duration of menstruation and an adenomyosis diagnosis after cesarean section were obtained by memory, which may have caused bias. Third, the sample size used to generate the data on subsequent pregnancies after treatment was small; therefore, the relationship between adenomyosis and pregnancy could not be assessed. Therefore, further prospective and large multi-center studies are needed in the future.

## Conclusions

Vaginal repair is a minimally invasive surgical procedure that can reduce postmenstrual spotting and repair the uterus to preserve fertility in patients with CSD. Based on the findings of this study, we are cautiously optimistic that adenomyosis might be an adverse factor for the healing of uterine incisions. Randomized double-blind controlled studies are needed to verify the positive correlation between myometrial repair and adenomyosis treatment.

## Data Availability

The datasets generated and/or analyzed during the current study are not publicly available due personal privacy but are available from the corresponding author on reasonable request.
